# Public health impact and return on investment of Belgium’s pediatric immunization program

**DOI:** 10.3389/fpubh.2023.1032385

**Published:** 2023-06-22

**Authors:** Justin Carrico, Claire E. Mellott, Sandra E. Talbird, André Bento-Abreu, Barbara Merckx, Jessica Vandenhaute, Damia Benchabane, Nicolas Dauby, Olivier Ethgen, Philippe Lepage, Jeroen Luyten, Marc Raes, Steven Simoens, Marc Van Ranst, Amanda Eiden, Mawuli K. Nyaku, Goran Bencina

**Affiliations:** ^1^RTI Health Solutions, Research Triangle Park, NC, United States; ^2^MSD, Brussels, Belgium; ^3^Department of Infectious Diseases, Centre Hospitalier Universitaire Saint-Pierre, Université Libre de Bruxelles (ULB), Brussels, Belgium; ^4^School of Public Health, ULB, Brussels, Belgium; ^5^Institute for Medical Immunology, ULB, Brussels, Belgium; ^6^Department of Public Health, Epidemiology and Health Economics, Faculty of Medicine, University of Liège, Liège, Belgium; ^7^SERFAN Innovation, Namur, Belgium; ^8^Paediatric Infectious Diseases, Hôpital Universitaire des Enfants Reine Fabiola and Université Libre de Bruxelles, Brussels, Belgium; ^9^Leuven Institute for Healthcare Policy, KU Leuven, Leuven, Belgium; ^10^Jessa Hospital, Hasselt, Belgium; ^11^Department of Pharmaceutical and Pharmacological Sciences, KU Leuven, Leuven, Belgium; ^12^Rega Institute for Medical Research, KU Leuven, Leuven, Belgium; ^13^Merck & Co., Inc., Rahway, NJ, United States; ^14^Center for Observational and Real-World Evidence, MSD, Madrid, Spain

**Keywords:** vaccination, model, cost-benefit analysis, national immunization program, expanded immunization program, infectious disease

## Abstract

**Objective:**

We evaluated the public health impact and return on investment of Belgium’s pediatric immunization program (PIP) from both healthcare-sector and societal perspectives.

**Methods:**

We developed a decision analytic model for 6 vaccines routinely administered in Belgium for children aged 0–10 years: DTaP-IPV-HepB-Hib, DTaP-IPV, MMR, PCV, rotavirus, and meningococcal type C. We used separate decision trees to model each of the 11 vaccine-preventable pathogens: diphtheria, tetanus, pertussis, poliomyelitis, *Haemophilus influenzae* type b, measles, mumps, rubella, *Streptococcus pneumoniae*, rotavirus, and meningococcal type C; hepatitis B was excluded because of surveillance limitations. The 2018 birth cohort was followed over its lifetime. The model projected and compared health outcomes and costs with and without immunization (based on vaccine-era and pre–vaccine era disease incidence estimates, respectively), assuming that observed reductions in disease incidence were fully attributable to vaccination. For the societal perspective, the model included productivity loss costs associated with immunization and disease in addition to direct medical costs. The model estimated discounted cases averted, disease-related deaths averted, life-years gained, quality-adjusted life-years gained, costs (2020 euros), and an overall benefit–cost ratio. Scenario analyses considered alternate assumptions for key model inputs.

**Results:**

Across all 11 pathogens, we estimated that the PIP prevented 226,000 cases of infections and 200 deaths, as well as the loss of 7,000 life-years and 8,000 quality-adjusted life-years over the lifetime of a birth cohort of 118,000 children. The PIP was associated with discounted vaccination costs of €91 million from the healthcare-sector perspective and €122 million from the societal perspective. However, vaccination costs were more than fully offset by disease-related costs averted, with the latter amounting to a discounted €126 million and €390 million from the healthcare-sector and societal perspectives, respectively. As a result, pediatric immunization was associated with overall discounted savings of €35 million and €268 million from the healthcare-sector and societal perspectives, respectively; every €1 invested in childhood immunization resulted in approximately €1.4 in disease-related cost savings to the health system and €3.2 in cost savings from a societal perspective for Belgium’s PIP. Estimates of the value of the PIP were most sensitive to changes in input assumptions for disease incidence, productivity losses due to disease-related mortality, and direct medical disease costs.

**Conclusion:**

Belgium’s PIP, which previously had not been systematically assessed, provides large-scale prevention of disease-related morbidity and premature mortality, and is associated with net savings to health system and society. Continued investment in the PIP is warranted to sustain its positive public health and financial impact.

## 1. Introduction

Vaccines are among the most cost-effective strategies to promote public health and prevent infectious diseases and associated morbidity, mortality, and disability (1). However, the comprehensive economic value of vaccines may be under recognized because effects such as reductions in disease complications, productivity gains for caregivers, and improvements in quality of life may not fully be reflected in the economic evidence (2). Further, as infections prevented from vaccination are not directly observable, immunization programs can fall victim to their own success. To ensure that sufficient resources are allocated for immunization programs, it is important that studies document the return on investment (ROI) that such programs offer.

Belgium has established a pediatric immunization schedule to protect against childhood vaccine-preventable infectious diseases. Belgium’s Superior Health Council recommends the following routine immunizations for children: diphtheria, tetanus, acellular pertussis, inactivated poliovirus, hepatitis B, and *Haemophilus influenza*e type b (hexavalent; DTaP-IPV-HepB-Hib); diphtheria, tetanus, acellular pertussis, and inactivated poliovirus (DTaP-IPV); measles, mumps, rubella (MMR); meningococcal type ACWY, pneumococcal conjugate (PCV); and rotavirus (3). Oversight of Belgium’s vaccination program takes place from federated entities, with separate authorities for Flanders, Wallonia, and Brussels. Vaccination coverage rates are near or above 90% for pediatric vaccines across Belgium, with slightly higher rates in Flanders as compared with Wallonia/Brussels regions (4). Certain childhood vaccines that are included in neighboring countries, such as meningococcal type B and varicella, are not part of the routine immunization program in Belgium.

The cost of vaccine acquisition and administration throughout life in 7 European countries was found to range from €443 to €3,395 per person (5). Despite the public health value of immunization programs, most European Union countries spend less than 0.5% of their healthcare budgets on immunization (6). When considering the investment in vaccination programs, studies in low- and middle-income countries have estimated favorable ROI for pediatric immunization programs (PIPs) (7, 8). Additionally, in the United States (US), an evaluation of the costs and the ROI of routine immunization in children for the 2017 birth cohort yielded a savings of $13.7 billion in direct healthcare costs and $55.1 billion to society, with benefit–cost ratios (BCRs) of 2.8 and 7.5, respectively (9). However, to our knowledge, the public health impact and value for money of PIPs as a whole have not previously been evaluated in high-income countries other than the US. Therefore, this study evaluated the public health and value for money of the Belgium PIP from both a healthcare-sector perspective and a societal perspective.

## 2. Methods

### 2.1. Model description

We developed a decision tree model in Microsoft Excel to analyze the public health impact and value for money of Belgium’s PIP. The model focused on 6 vaccines routinely administered from birth through age 10 years in Belgium: hexavalent (DTaP-IPV-HepB-Hib), DTaP-IPV, MMR, PCV, rotavirus, and meningococcal type C. While meningococcal type ACWY vaccination is currently recommended by the Superior Health Council (3), it was not included in Belgium vaccination schemes at the time of the analysis. Meningococcal type C vaccination, which was recommended prior to the meningococcal type ACWY recommendation, was therefore modeled. Adolescent (e.g., human papilloma virus, DTaP booster) and adult vaccines were not modeled. Because reporting of hepatitis B incidence in Belgium improved after the introduction of a hepatitis B vaccine, pre-vaccine incidence was likely significantly underreported; therefore, the impact of the immunization program on hepatitis B disease incidence and costs was conservatively omitted (10–12).

The 2018 Belgian birth cohort was modeled and followed from birth to age 100 years. Two analytical scenarios were constructed: one in which routine pediatric immunization occurred and one in which no immunization occurred. The incidence of modeled diseases reflected current rates of disease in the scenario with pediatric immunization, whereas incidence in the counterfactual scenario without immunization was assumed to reflect pre-vaccine levels. Thus, reductions in disease incidence were estimated from observed incidence data rather than mathematically modeled from vaccination coverage and effectiveness data and observed reductions in disease incidence were assumed to be fully attributed to vaccination. This model structure and analytic framework was previously used to analyze the PIP in the US Carrico et al. (9).

Separate decision trees were used to estimate the incidence and costs of each pathogen covered by Belgium’s PIP (i.e., diphtheria, tetanus, pertussis, poliomyelitis, *H influenzae* type b, measles, mumps, rubella, *Streptococcus pneumoniae*, rotavirus, and meningococcal type C disease). Disease outcomes and costs were calculated every month from birth until age 12 months and then for every year thereafter. Each cycle, we subtracted the number of individuals who died from all-cause mortality from the birth cohort, and the remaining individuals progressed to the next cycle. Age-specific all-cause mortality, disease incidence, disease management costs, and probability of different clinical outcomes were used to parameterize the decision tree for each disease. For each disease, the total cases of a given severity were calculated by multiplying the likelihood of different clinical outcomes (including death) by the total number of expected cases. Total disease management costs were calculated by multiplying costs for a given clinical outcome by the number of clinical outcomes among the total number of disease cases. Surviving individuals who developed long-term complications for some diseases accumulated disability-related costs and quality-of-life reductions, which were discounted over their remaining lifetimes.

Vaccination costs for the birth cohort were modeled using vaccination coverage estimates and assumed timing of administering vaccine doses according to Belgium’s recommended pediatric immunization schedule (3). Vaccination coverage estimates were used for estimation of vaccination costs only, as disease cases were estimated using observed incidence data.

The model compared lifetime health outcomes and costs between the PIP scenario and a scenario without any immunization. Analyses were conducted from both a healthcare-sector perspective and a societal perspective. Health outcomes and costs were discounted at an annual rate of 1.5 and 3.0%, respectively, in line with Belgian recommendations for economic evaluation (20). Costs were inflated to 2020 euros using the consumer price index for Belgium (21).

### 2.2. Immunization program costs

We calculated the costs of the immunization program based on the 2021 immunization schedule and the most recent vaccine coverage data available at the time of the analysis, which were from 2019–2020 (Table 1). For simplicity reasons, we looked at vaccine coverage for Belgium as a whole for our model. This was done by obtaining vaccination coverage from the Flanders, Wallonia, and Brussels regions (4, 13, 15); then a weighted average coverage for Belgium was calculated using the 2020 total population size estimates among ages less than 10 years for each region from Statbel, the Belgian statistical office (14). The number of people in the birth cohort receiving each dose of recommended vaccines in Belgium’s PIP was calculated by multiplying the vaccination coverage for each dose by the size of the birth cohort at each age of recommended vaccination. No adjustments were made to account for a proportion of children receiving the vaccine later than the recommended age.

**Table 1:** Childhood immunization schedule, coverage estimates, and vaccine acquisition costs.

Vaccine	Age at vaccination	Coverage (% fully vaccinated)^a^	Acquisition cost per dose ^b^
DTaP-IPV^c^	5 years	84.0%	€30.08
Hexavalent (DTaP-IPV-HepB-Hib)	2, 3, 4, 15 months	93.9%	€53.66
Meningococcal type C	15 months	92.1%	€35.63
MMR	12 months, 10 years	83.0%	€25.19
PCV	2, 4, 12 months	93.8%	€74.55
Rotavirus	2 doses: 2, 3 months	85.8%	€68.80^d^
	3 doses: 2, 3, 4 months		

DTaP-IPV = diphtheria, tetanus, acellular pertussis, and inactivated poliovirus; DTaP-IPV-HepB-Hib = diphtheria, tetanus, acellular pertussis, inactivated poliovirus, hepatitis B, and H influenzae type b (hexavalent); MMR = measles, mumps, rubella; PCV = pneumococcal conjugate vaccine.

a Vaccine coverage values are a weighted average of the vaccine coverage rates and population proportion among ages less than 10 years for Flanders, Wallonia, and Brussels (4,13, 14). All coverage data were from 2019–2020, except for DTaP-IPV coverage in Wallonia and Brussels, which was obtained from 2015 data (15). For vaccines with multiple doses, the coverage rate shown is the percentage of children that have received the full vaccination series. Weighted vaccination coverage rates for the first, second, and third doses of hexavalent vaccine were 98.4%, 98.0%, and 97.5%, respectively; 96.0% for the first dose of MMR; 97.3 and 96.9% for the first and second dose of PCV, respectively; and 88.9% for the first dose of rotavirus (2-dose or 3-dose) (4). 2019–2020 coverage data did not specify the percentage of those receiving the 2-dose and 3-dose rotavirus series; thus, among those vaccinated, 86.2 and 13.8% were estimated to receive the 2-dose and 3-dose series, respectively, from 2015–2016 data (16, 17).

b Values for vaccine list price per dose are from RIZIV/INAMI and CBIP (18, 19). Vaccine list prices assumed use of vaccines in Belgium’s tender, including Tetravac for DTaP-IPV, Hexyon for hexavalent vaccine, NeisVac-C for meningococcal type C, M-M-RVaxPro for MMR, and Prevnar-13 for PCV. A combination of 2-dose and 3-dose rotavirus vaccine was also assumed.

c DTaP-IPV at 5 years is a booster recommended for the whole population.

d The cost shown is a weighted average between the 2-dose vaccine cost (€71.48) and 3-dose vaccine cost (€51.82). Among those receiving rotavirus vaccine, 86.2 and 13.8% were estimated to receive the 2-dose and 3-dose series, respectively (16, 17).

Vaccine acquisition costs were obtained from public vaccine prices from the Belgian National Institute for Health and Disability Insurance and the Belgian Pharmacotherapeutic Information Center (18, 19). Costs of vaccine administration were incurred per vaccination visit, with vaccination visits distributed among well-child clinics, pediatricians, and general practitioners (22, 23). Vaccine-related adverse events incurred costs associated with outpatient visits and hospitalizations (23–25).

Travel and productivity loss costs for time spent by a caregiver to bring their child to receive each vaccine were included in the societal perspective. Hourly costs of caregiver time were calculated from 2018 Statbel estimates of annual gross salary for Belgium residents. Travel costs were assumed to be €5 per vaccination visit. Fifty percent of the travel and productivity loss costs for each physician visit during which immunizations were given were attributed to the cost of the immunization program. Previous economic analyses presented wide variations in approaches for capturing indirect costs associated with child vaccination; therefore, this parameter was tested in sensitivity analysis using the widest range (0–100%).

### 2.3. Disease incidence

We considered incidence data before and after each vaccine was routinely recommended for each vaccine-preventable disease (Table 2). Pre-vaccine incidence was obtained using published incidence estimates for Belgium or calculated using published annual case estimates and Belgium population data for the same period (26–37). Average incidence over multiple years was used if available to account for fluctuations in annual disease incidence. Pre-vaccine incidence rates from other European countries were used for Belgium when local data were unavailable, which included Poland for tetanus, pertussis, and rubella and France for *H influenzae* type b (38–45). Our analysis assumed that that any observed reduction in disease incidence after vaccine introduction was fully caused by vaccination within the population. This approach of using pre-vaccine and vaccine-era incidence implicitly captured the impact that vaccination coverage, efficacy, and waning have on disease incidence at the population level. Thus, vaccination coverage rates used to estimate immunization program costs were not used to estimate reductions in disease incidence due to immunization. This approach was a simplification of the complex dynamics that drive infectious disease transmission, which also include factors such as changes in individual behaviors, sanitation and hygiene, epidemiological circumstances, and exchanges and interactions with other countries and populations. Nonetheless, there is a wide consensus that lower incidences from pediatric infectious diseases are largely caused by vaccine introduction within the population (46–50). To demonstrate the influence of this assumption, we conducted scenario analyses in which lower pre-vaccine incidences and higher vaccine-era incidences were separately tested.

**Table 2:** Pre–vaccine era and vaccine-era disease incidence estimates.

Pathogen	Disease incidence per 100,000 by age group					
	<1 y	1–4 y	5–9 y	10–19 y	20–64 y	≥65 y
Diphtheria (26, 27, 51)						
Without PIP (pre-vaccine)	8	8	8	8	8	8
With PIP (vaccine era)	<1	<1	<1	<1	<1	<1
*H influenzae* type b (27, 38, 53)						
Without PIP (pre-vaccine)^a^^,^^b^	16–69	6–34	NA	NA	NA	NA
With PIP (vaccine era)	<1	<1	<1	<1	<1	<1
Measles (27, 28, 54)						
Without PIP (pre-vaccine)^a^	1,204	9,451	6,309	208–1,326	15	15
With PIP (vaccine era)	21	8	4	2	1–2	1
Meningococcal type C (27, 29, 30, 60)						
Without PIP (pre-vaccine)^a^	16	5	1	1	<1–1	<1
With PIP (vaccine era)	1	<1	<1	<1	<1	<1
Mumps (27, 28, 55)						
Without PIP (pre-vaccine)^a^	282	5,430	4,859	416–1,093	77	77
With PIP (vaccine era)^a^	1	3	4	4–6	1–4	1
Pertussis (27, 39, 56)						
Without PIP (pre-vaccine)^a^	664	739–1,041	408	10–89	10	10
With PIP (vaccine era)^a^	58	20	21	16–27	5	5
Polio (27, 31, 58)						
Without PIP (pre-vaccine)	5	5	5	5	5	5
With PIP (vaccine era)	0	0	0	0	0	0
Rotavirus (35–37, 65, 66)						
Hospitalizations^c^						
Without PIP (pre-vaccine)	2,372	755	NA	NA	NA	NA
With PIP (vaccine era)	300	143	NA	NA	NA	NA
Outpatient visits^c^						
Without PIP (pre-vaccine)	3,964	3,964	NA	NA	NA	NA
With PIP (vaccine era)	798	798	NA	NA	NA	NA
Rubella (27, 40–44)						
Without PIP (pre-vaccine)	200	110–329	897	62–382	1–11	0
With PIP (vaccine era)	<1	<1	<1	<1	<1	<1
*S pneumoniae*						
Invasive pneumococcal disease (32, 33, 61, 62)						
Without PIP (pre-vaccine)^a^	156	42–156	10	5–10	5–15	26–80
With PIP (vaccine era)^a^	51	16–51	5	2–5	2–5	9–28
Pneumonia hospitalizations (34, 63)^d^						
Without PIP (pre-vaccine)^a^	716	563	130	17–39	12–40	113–372
With PIP (vaccine era)^a^	652	498–547	127	16–36	11–37	108–357
Pneumonia outpatient visits (33, 34)^d^						
Without PIP (pre-vaccine)^a^	961	961	128	43–128	43–163	184–411
With PIP (vaccine era) ^a^	896	896–945	125	42–125	42–161	179–396
Acute otitis media (34, 64)^d^						
Without PIP (pre-vaccine)^a^^,^^e^	5,968	5,968	1,920	525–1,920	NA	NA
With PIP (vaccine era)^a^^,^^e^	1,273	1,273-1,604	541	148–541	NA	NA
Tetanus (27, 45, 59)						
Without PIP (pre-vaccine)	1	1	2	1–2	<1–1	1
With PIP (vaccine era)	0	0	0	0	0	0

NA = not applicable; PIP = pediatric immunization program. Reporting of hepatitis B incidence improved after the introduction of a hepatitis B vaccine, so pre-vaccine incidence is likely significantly underreported. Therefore, pre-vaccine incidence was assumed to be the same as vaccine-era incidence.

a A range indicates that incidence varies by age group within the presented range.

b Pre-vaccine incidence was not reported for ages 5+ years.

c Incidence was not modeled for ages 5+ years.

d All-cause pneumonia and otitis media incidence rates were adjusted to account for the percentage of cases that were due to *S. pneumoniae* (74–76).

e Incidence was not modeled for ages 18+ years.

Vaccine-era incidence for each disease was obtained from the most recent surveillance data from the European Centre for Disease Prevention and Control, calculated from published annual case estimates and Belgium population data, or derived from published vaccination impact and effectiveness studies (32, 33, 51–66). Average incidence over the most recent 5 years of surveillance data were used if available.

For both pre-vaccine and vaccine-era incidence, age-specific incidence was used when available. Incidence was not adjusted for underreporting; thus, disease underreporting was only captured in incidence estimates if the underlying data source adjusted for underreporting. Additional details regarding the date of vaccination program initiation for each disease, as well as sources and time periods for pre-vaccine and vaccine-era incidence estimates, are available in the Supplementary Material.

### 2.4. Healthcare-sector disease costs

To capture the costs from disease cases, the model considered disease-specific case severity distributions, disease case-fatality rates, direct medical costs per case, and costs for management of long-term sequelae, with estimates based on the published literature (Table 3; Supplementary Material Tables A-4 to A-17). Case-fatality rates and costs for disease management were intended to reflect the current standard of care for managing vaccine-preventable disease cases.

**Table 3:** Outcomes and direct medical costs by disease.

Pathogen	Percentage of cases resulting in hospitalization/severe case	Percentage of cases resulting in death	Cost per hospitalization/severe case	Cost per outpatient case/visit
Diphtheria	100.0%	10.0%	€2,935	NA
*H influenzae* type b	50–100%	3.8%	€2,070–€10,745	€45–€948
Measles^a^	0.1–7.2%	0.1%	€484–€172,610	€85–€192
Meningococcal type C	74.6%	7.1–35.7%	€6,457–€11,457	€108
Mumps^b^	11.5–48.5%	0.0%	€1,541–€4,901	€127
Pertussis	7.8%	<0.1%	€976–€3,414	€21–€26
Polio^c^	100.0%	2.0%	€88,713	NA
Rotavirus	14.7–36.4%^d^	<0.1%	€1,063	€107
Rubella^e^	0.0–30.0%	0.0%^f^	€40,947	€52
*S pneumoniae*				
Invasive pneumococcal disease	100.0%	0.0–23.4%	€3,167–€10,745	NA
*S pneumoniae* pneumonia	18.7–57.4%^g^	0.0–22.4%	€3,758–€6,147	€88–€948
*S pneumoniae* acute otitis media	0.1–2.5%	0.0%	€4,083–€4,553	€91–€371
Tetanus	100.0%	15.0%	€76,988	NA

NA = not applicable. A range indicates that a parameter varies by age group and/or clinical outcome within the presented range. Values and sources for all parameters presented in this table are presented in additional detail in the Supplementary Material.

a Hospitalizations and outpatient visits were not defined in the measles case severity distribution. Therefore, the information presented for hospitalizations reflects encephalitis and pneumonia cases, while the information for outpatient visits reflects otitis media and other uncomplicated cases.

b Hospitalizations and outpatient visits were not defined in the mumps case severity distribution. Therefore, the information presented for hospitalizations and outpatient visits reflects complicated and uncomplicated cases, respectively.

c Hospitalizations and outpatient visits were not defined in the polio case severity distribution. Therefore, the information presented for hospitalizations and outpatient visits reflects paralytic and nonparalytic cases, respectively.

d Calculated by dividing the incidence rate for rotavirus hospitalizations by the total incidence rate for rotavirus (including hospitalizations, outpatient visits, and non–medically attended cases).

e Hospitalizations and outpatient visits were not defined in the rubella case severity distribution. Therefore, the information presented for hospitalizations and outpatient visits reflects complicated and uncomplicated cases, respectively.

f 10.6 and 0.4% of infants born with congenital rubella syndrome die within the first and second year of life, respectively (77).

g Calculated by dividing the incidence rate for S. pneumoniae hospitalizations by the total incidence rate for S. pneumoniae (including both hospitalizations and outpatient visits).

### 2.5. Productivity losses due to disease

The human capital approach was applied to calculate the value of time loss due to acute disease, long-term complications, and disease-related mortality (24, 67, 69). Mean workdays lost per disease case (Supplementary Material Table A-19) were multiplied by daily productivity, which was calculated from annual gross salary, to capture productivity losses due to acute disease. Age-specific annual productivity and life expectancy were used to calculate productivity losses associated with long-term complications and disease-related deaths. The percentage reduction in annual productivity for long-term complications was assumed to be equal to the percentage reduction in health-related utility weights associated with the complication (Supplementary Material Table A-20).

### 2.6. Quality-of-life impacts

Vaccinated individuals may experience adverse-event–related quality-of-life impacts. To calculate quality-adjusted life-years (QALYs) lost because of adverse events, the number of vaccine-related adverse events experienced by the birth cohort was multiplied by adverse event–specific disutilities and their associated durations (Supplementary Material Table A-3). Disease impact on quality of life was calculated using disease-specific disutilities and their associated durations (Supplementary Material Table A-18) (9).

### 2.7. Outcomes

Vaccine acquisition costs associated with the Belgium’s PIP were calculated by multiplying the number of vaccine doses administered by acquisition costs per dose. Vaccine administration costs were calculated by multiplying the expected number of vaccination visits by the administration cost per visit, allowing for multiple vaccines to be given at a single physician visit according to the immunization schedule. Vaccine-related adverse event incidence per dose, cost per adverse event, and number of vaccine doses administered were multiplied to calculate adverse event costs. For the societal perspective only, costs for travel and productivity loss for caregiver time for vaccination visits were calculated using the expected number of vaccination visits, travel costs per visit, and time loss costs per visit.

The number of disease cases for the 11 modeled pathogens for the 2018 birth cohort was calculated using incidence rates from the pre-vaccine and vaccine eras. Clinical outcomes by severity were then calculated, including the number of cases with lifelong sequelae and disease-related deaths.

Costs of disease for the 11 modeled pathogens were calculated with the current PIP and for comparison with the cost of disease if the PIP were discontinued and incidence were to revert to pre-vaccine levels. Specifically, the cost per disease outcome (by severity) was multiplied by the number of clinical outcomes in the pre-vaccine and vaccine eras. Costs of long-term sequelae were calculated using an annual cost, discounted over the duration of the sequela or over the cohort’s remaining lifetime. For the societal perspective only, productivity loss for disease-related deaths was calculated as the number of deaths at each age by the discounted lifetime productivity at that age.

QALYs were calculated similarly to costs, with the disutility and duration value per disease or adverse event outcome multiplied by the number of clinical outcomes. The monetary value of total QALYs gained was calculated by multiplying total QALYs gained by a willingness-to-pay threshold of €39,500 per QALY gained, which is roughly the gross domestic product *per capita* in Belgium (70, 68).

### 2.8. Analyses

The financial BCR of the Belgium PIP was calculated for each perspective by dividing the costs of disease cases averted by the net vaccination costs, as done in similar previous analyses (9, 67). This was calculated according to the equation:


$$BCR=\frac{\sum_{t=1}^{T}\frac{B_{t}}{{\left(1+r\right)}^{t}}}{\sum_{t=1}^{T}\frac{C_{t}}{{\left(1+r\right)}^{t}}\;},$$

where *B_t_* represents the annual direct costs savings from cases prevented by vaccination in year *t*, *C_t_* represents the annual direct costs of vaccination in year *t*, *t* represents the year of the analysis, *r* represents the discount rate, and *T* represents the number of years in the time horizon.

For the societal perspective, *B_t_* also includes productivity losses from disease-related morbidity and mortality averted, and *C_t_* also includes productivity losses owing to vaccination visits.

Cases averted and deaths averted because of the PIP were also calculated. QALYs gained were calculated as the difference in QALYs lost between the scenarios with and without the Belgium PIP. The monetary value of QALYs gained was not included in the financial BCR for the base-case analysis and is instead reported as a standalone outcome, as a formal cost–benefit analysis was not conducted.

#### 2.8.1. Scenario analyses

In addition to base-case analyses, scenario analysis was conducted to assess the robustness of model results to changes in key assumptions. Scenarios considered variations to the following assumptions: (1–2) 10 and 20% reduction in pre-vaccine disease incidence; (3–4) 10 and 20% increase in vaccine-era disease incidence; (5–6) 20 and 40% reduction in vaccine acquisition costs to reflect vaccine tender prices; (7) 20% increase in pre-vaccine incidence underreporting for all diseases except meningococcal type C disease; (8) 20% increase in vaccine-era incidence underreporting for measles, mumps, pneumococcal disease, and rotavirus; (9–10) 20% increase and decrease in healthcare-sector disease-related costs; (11–12) 20% increase and decrease in health disutility values; (13) exclusion of productivity losses due to disease-related mortality; (14) 10-year time horizon (versus lifetime time horizon in base-case analysis); (15) inclusion of the economic value of QALYs gained in the benefits variable of the financial BCR calculation; (16) 20% reduction in pre-vaccine disease incidence and 20% reduction in vaccine acquisition costs (combination of Scenario 2 and Scenario 5); and (17) 20% increase in vaccine-era disease incidence and 20% reduction in vaccine acquisition costs (combination of Scenario 4 and Scenario 5).

## 3. Results

### 3.1. Health outcomes without and with immunization

For the 2018 Belgian birth cohort of 117,800 individuals, there were an estimated 265,000 preventable disease cases without the PIP, resulting in 508 disease-related deaths, 10,300 life-years (LYs) lost, and 11,000 QALYs lost because of disease-related morbidity and mortality. When modeled with the Belgium PIP, there were an estimated 39,000 vaccine-preventable disease cases and 294 disease-related deaths, with 3,300 LYs and 3,100 QALYs lost. Therefore, the Belgium PIP was associated with approximately 226,000 disease cases averted, 214 disease-related deaths averted, 7,100 LYs gained, and 8,000 QALYs gained (Table 4).

**Table 4:** Base-case health outcome results, overall and by disease.

Pathogen	Cases averted	Premature deaths averted	LYs gained	QALYs gained
Diphtheria	460	46	1,532	1,348
*H influenzae* type b	116	4	207	306
Measles	84,725	68	3,071	3,425
Meningococcal type C	67	9	387	414
Mumps	59,938	0	0	261
Pertussis	7,089	3	134	225
Polio	294	6	196	250
Rotavirus^a^	23,822	1	46	133
Rubella	7,989	<1	4	34
*S pneumoniae* ^b^	41,768	68	1,221	1,357
Tetanus	56	8	264	234
Total	226,324	214	7,062	7,988^c^
Total (Undiscounted)	248,765	383	14,472	14,500^c^

LY = life-year; QALY = quality-adjusted life-year. Health outcomes are not shown for hepatitis B as cases averted are 0.

a Rotavirus total “cases” are reported as a sum of rotavirus-related hospitalizations, emergency department visits, outpatient visits, and non–medically attended cases. The “cases” sum may be an overestimate of total rotavirus cases in the population, as some events may have multiple rotavirus-related visits.

b Total *S. pneumoniae* “cases” are reported as a sum of cases of invasive pneumococcal disease, pneumococcal pneumonia, and acute otitis media.

c Total QALYs gained because of the Belgium PIP are 7,966 (discounted; 14,477, undiscounted) when QALYs lost from vaccine-related adverse events are included.

The PIP’s impact on disease morbidity and mortality varied by disease, with the most cases averted for measles (84,725 cases averted), mumps (59,938 cases averted), and pneumococcal disease (41,768 cases averted). Disease-related deaths averted, LYs gained, and QALYs gained were highest for measles (68 deaths averted, 3,071 LYs gained, and 3,425 QALYs gained), pneumococcal disease (68 deaths averted, 1,221 LYs gained, and 1,357 QALYs gained), and diphtheria (46 deaths averted, 1,532 LYs gained, and 1,348 QALYs gained; Table 4).

Table 4 presents undiscounted incremental health outcomes associated with the Belgium PIP. For each disease evaluated, each associated vaccine in the Belgium PIP reduced the number of cases from approximately 51% for pneumococcal disease to 100% for tetanus and polio. A reduction in cases of more than 90% was achieved for 8 of the 11 pathogens evaluated.

### 3.2. Cost outcomes without and with immunization

Without the Belgian PIP, lifetime discounted societal disease-related costs for the birth cohort were an estimated €515 million, 43% (€222 million) owing to productivity losses from disease-related mortality, 33% (€169 million) owing to healthcare-sector costs to treat cases of disease, and the remainder owing to productivity losses from cases of disease and long-term sequelae (€124 million). Lifetime societal disease-related costs were €125 million when diseases were modeled with the Belgium PIP, including €43 million in healthcare-sector costs to treat cases of disease, €15 million in productivity losses due to cases of disease and long-term sequelae, and €67 million in productivity losses due to disease-related mortality.

**Figure 1:**
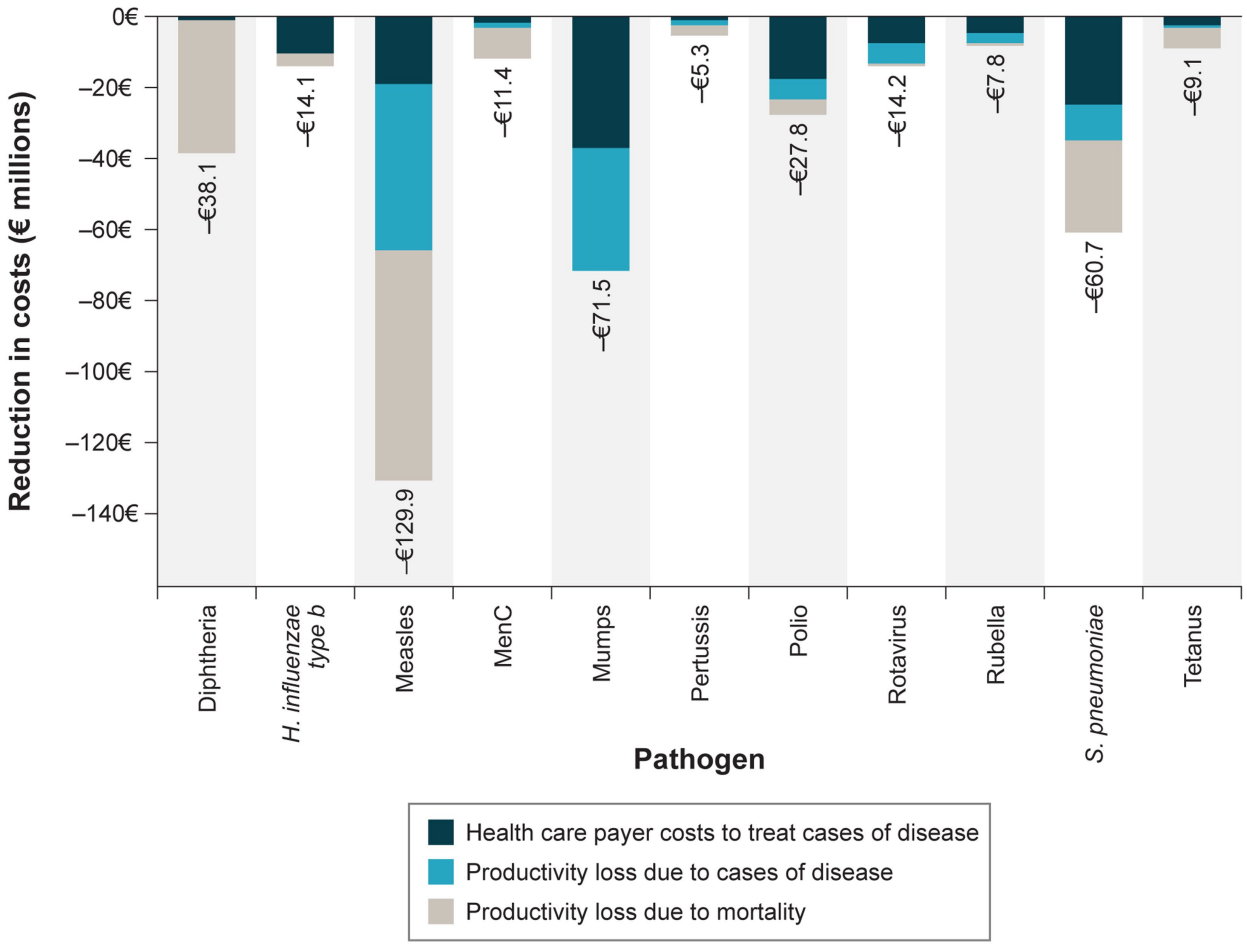
Societal disease-related costs averted by the Belgium PIP by disease and cost category. MenC = meningococcal type C; PIP = pediatric immunization program. Costs are presented in 2020 euros discounted at an annual rate of 3%.

Therefore, discounted societal disease-related cost savings due to Belgian PIP totaled €390 million, with the highest savings from averted cases of measles, mumps, and pneumococcal disease (Figure 1). For disease-related costs averted, healthcare-sector costs to treat individuals with acute cases of disease and long-term sequelae accounted for 32% of the savings, while the remaining costs averted were from productivity losses due to cases of disease and long-term sequelae (28%) and productivity loss due to mortality (40%; Table 5).

**Table 5:** Return on investment for the Belgium PIP program compared with no PIP.

Incremental results	Healthcare-sector perspective (€ millions)	Societal perspective (€ millions)
Incremental vaccination costs	€91	€122
Acquisition	€78	€78
Administration	€11	€11
Adverse events	€1	€1
Productivity loss (time and travel) for vaccination	−	€31
Disease-related costs averted	€126	€390
Disease treatment	€126	€126
Productivity loss due to disease	−	€110
Productivity loss due to disease-related mortality	−	€155
Total costs averted	€35	€268
Financial benefit–cost ratio	1.4	3.2
Value of QALYs gained^a^	−	€315

PIP = pediatric immunization program; QALY = quality-adjusted life-year. Costs are presented in 2020 euros discounted at an annual rate of 3%.

a The value of QALYs gained is calculated by multiplying the total QALYs gained with the PIP by a willingness-to-pay threshold of €39,537, which is the gross domestic product *per capita* in Belgium in 2020 (68). The World Health Organization recommends a willingness-to-pay threshold range of 1 to 3 times the per-capita gross domestic product (69). This value was not included in the calculation of the financial benefit–cost ratio for the base-case analysis.

Immunization of the 2018 Belgian birth cohort resulted in €122 million in discounted societal vaccination costs, with most costs associated with vaccine acquisition (64%), administration (9%), and caregiver time and travel (25%); adverse events constituted a small proportion of costs (1%). Vaccination costs were significantly outweighed by disease-related costs averted, resulting in net savings of €268 million and a financial BCR of 3.2 from the societal perspective. This BCR indicates that every €1 invested in the Belgian PIP is expected to result in over €3 in savings to society. When productivity losses were excluded in the healthcare-sector perspective, a financial BCR of 1.4 was observed. Although the economic value of QALYs gained because of the Belgian PIP was not included in the financial BCR calculation in the base-case analysis, the total QALYs gained was valued at approximately €315 million when a willingness-to-pay threshold of €39,500 per QALY gained was considered (70, 68).

### 3.3. Scenario analyses

When key assumptions and analysis settings were modified in scenario analyses, the societal BCR for the Belgium PIP was lowest when productivity losses due to disease-related mortality were excluded (BCR = 1.9). Scenarios related to input data assumptions demonstrated that QALYs gained were most impacted by variations in assumptions for disease incidence and disutility values and that BCRs were most impacted by variations in assumptions for incidence and direct medical costs per case (Table 6). When the economic value of QALYs gained was included in the BCR calculation, the societal BCR substantially increased (BCR = 5.8). The impact of changes in pre-vaccine and vaccine era disease incidence on the societal BCR was mitigated when vaccine acquisition costs were reduced to reflect tender prices.

**Table 6:** Results for scenarios considering variations in key input values.

Scenario	Healthcare-sector BCR	Societal BCR	Total QALYs gained
Base case	1.4	3.2	8,000
Scenario 1: 10% reduction in pre-vaccine disease incidence	1.2	2.8	6,900
Scenario 2: 20% reduction in pre-vaccine disease incidence	1.0	2.4	5,800
Scenario 3: 10% increase in vaccine-era disease incidence	1.3	3.1	7,700
Scenario 4: 20% increase in vaccine-era disease incidence	1.3	3.0	7,400
Scenario 5: 20% reduction in acquisition cost for all vaccines except rotavirus^a^	1.6	3.6	8,000
Scenario 6: 40% reduction in acquisition cost for all vaccines except rotavirus^a^	1.9	4.0	8,000
Scenario 7: 20% increase in pre-vaccine incidence underreporting for all diseases except meningococcal type C disease	1.5	3.5	9,100
Scenario 8: 20% increase in vaccine-era incidence underreporting for measles, mumps, pneumococcal disease, and rotavirus	1.3	3.0	7,400
Scenario 9: 20% increase in healthcare-sector disease-related costs	1.7	3.4	8,000
Scenario 10: 20% reduction in healthcare-sector disease-related costs	1.1	3.0	8,000
Scenario 11: 20% increase in health disutility values	1.4	3.2	8,300
Scenario 12: 20% reduction in health disutility values	1.4	3.2	7,600
Scenario 13: exclusion of productivity losses due to disease-related mortality	1.4	1.9	8,000
Scenario 14: 10-year analysis time horizon^b^	1.1	2.4	5,600
Scenario 15: inclusion of the economic value of QALYs gained in the societal BCR calculation	1.4	5.8	8,000
Scenario 16: 20% reduction in pre-vaccine disease incidence and 20% reduction in acquisition cost for all vaccines except rotavirus^a^^,^^c^	1.2	2.6	5,800
Scenario 17: 20% increase in vaccine-era disease incidence and 20% reduction in acquisition cost for all vaccines except rotavirus^a^^,^^d^	1.5	3.3	7,400

BCR = benefit–cost ratio; QALY = quality-adjusted life-year.

a The price for rotavirus vaccine was not varied because it is not included in the tender for Belgium’s PIP.

b In this scenario, the birth cohort was only modeled through 10 years of age.

c Multi-way scenario combining Scenario 2 and Scenario 5.

d Multi-way scenario combining Scenario 4 and Scenario 5.

## 4. Discussion

This analysis found that Belgium’s PIP averted 226,000 cases and 214 premature deaths for the 2018 birth cohort over its lifetime, resulting in net savings from both a societal perspective (BCR = 3.2) and healthcare-sector perspective (BCR = 1.4). Public health achievements likely attributable to the PIP included the reduction in incidence of diphtheria, *H influenzae* type b, polio, rubella, and tetanus to negligible levels (<1 case per 100,000 population annually) and a reduction of >90% in incidence for 8 of the 11 pathogens covered by the PIP. When key input values were varied in scenario analyses, financial BCRs from the healthcare-sector and societal perspectives remained at or above 1, highlighting the robustness of the ROI for the Belgium PIP to the health system and society.

Previous cost–benefit analyses have evaluated the US PIP, with BCRs ranging from 2.8 to 3.0 and 7.5 to 10.1 from healthcare-sector and societal perspectives, respectively (9, 67). Analyses of immunization programs in low- and middle-income countries have also estimated a positive ROI for pediatric immunization (7, 8). Estimated BCRs for Belgium from the current analysis were lower than those estimated in the US. Differences in BCRs across countries could be explained by differences in healthcare costs, disease epidemiology, and magnitude and consistency of vaccination uptake, among other factors. Despite the lower BCRs relative to the US estimated in this study, pediatric immunization has been consistently demonstrated to provide significant economic value to society.

Results for the Belgium PIP found that averted cost of illness was greatest for averted cases of measles (i.e., €129.9 million), leading to nearly one-third of the total cost savings; this was aligned with prior findings that vaccination for the prevention of measles yielded the greatest costs savings across childhood immunization programs globally (~37% of total averted costs during the 2001–2020 period) (7). The visibility of the value of pediatric immunization is increasingly important, as the COVID-19 pandemic has significantly disrupted immunization rates in some European countries (71), creating a risk of resurgence of vaccine-preventable diseases. Outbreaks of measles have been increasingly documented since the start of the pandemic, and global measles cases have increased 79% year over year in the first 2 months of 2022 (72).

This analysis included limitations that must be noted. First, this analysis did not directly account for other public health improvements in addition to the Belgium PIP that have occurred in parallel with vaccine introductions over time and likely also contribute to reducing the burden of vaccine-preventable disease. In other words, the analysis framework assumed that all reductions in disease incidence were fully attributable to vaccination. Because of the limitations introduced by our incidence approach, we conducted scenarios with decreased pre-vaccine incidence (−10% and − 20%) and increased vaccine-era incidence (+10% and + 20%) across all modeled diseases to test the robustness of our analysis results. The PIP remained a valuable investment under such scenarios. Further, costs of adolescent and adult vaccines, as well as the proportion of disease reduction attributable to these vaccines, were not considered. A positive return on investment for the Belgium PIP was observed in a scenario analysis restricting the analysis time horizon to only model ages 0 to 10 years, indicating that the PIP was a valuable investment even when disease reductions that may be partially attributable to adolescent and adult vaccines were omitted. The human capital approach was used to estimate productivity losses due to mortality, consistent with previous economic evaluations of immunization programs (7, 9, 67, 73). Use of other methods of productivity loss estimation (e.g., friction cost approach) would result in reduced productivity losses due to mortality. These limitations may have allowed for overestimation of the impact of pediatric immunization on the reduction of vaccine-preventable disease morbidity, mortality, and economic burden. However, the analysis did not capture the impact of pediatric immunizations on groups outside the modeled cohort through herd immunity or other extended benefits of vaccination, such as increased educational attainment, increased productivity, reduced household financial risk, and reduced use of antimicrobials for vaccine-preventable diseases. These omissions may have contributed to underestimation of the disease impact and value of pediatric immunization. This analysis also considered public prices of vaccines, which are higher than tendered prices. Scenario analyses considering hypothetical reductions in vaccine prices improved the ROI for the PIP. Therefore, use of public prices in the analysis underestimates the value for money of the PIP.

Data limitations were also significant, as current estimates of disease outcomes (e.g., case-fatality ratios) and disease treatment costs could not be obtained for diseases that have been mostly eliminated. When such data unavailability was encountered, data from countries other than Belgium and/or assumptions supported by subject matter experts were applied. Scenario analyses demonstrated that the ROI for the PIP was robust to variations in assumptions for case-fatality ratios and healthcare-sector costs associated with disease cases. Disease-underreporting factors were conservatively not applied in the pre-vaccine era or vaccine era, which may underestimate the disease impact and value of pediatric immunization, as disease underreporting is likely more significant in the pre-vaccine era.

In conclusion, this is the first analysis to systematically assess the public health and economic effects of a European country’s PIP. The analysis estimated that routine pediatric immunization in Belgium averted more than 220,000 disease cases and 200 disease-related deaths for the 2018 birth cohort. Every €1 invested in pediatric immunization resulted in a €3 ROI from the societal perspective. Therefore, Belgium’s PIP brings large-scale prevention of morbidity and mortality and an associated reduction in costs, highlighting the value of pediatric immunization.

## Data availability statement

The original contributions presented in the study are included in the article/Supplementary material. Further inquiries can be directed to the corresponding author.

## Ethics statement

Ethical review and approval was not required for the study on human participants in accordance with the local legislation and institutional requirements. Written informed consent for participation was not required for this study in accordance with the national legislation and the institutional requirements.

## Author contributions

JC, CM, ST, and MN participated in the conception, design, and planning of the study, in the acquisition and analysis of the data, in the interpretation of the results, and in the drafting of the manuscript and critically reviewing and revising it for important intellectual content. AB-A and DB participated in the acquisition and analysis of the data, in the interpretation of the results, and in the drafting of the manuscript and critically reviewing and revising it for important intellectual content. BM and JV participated in the conception, design, and planning of the study, in the analysis of the data, in the interpretation of the results, and in critically reviewing and revising the manuscript for important intellectual content. ND, PL, JL, and OE participated in the analysis of the data, in the interpretation of the results, and in critically reviewing and revising the manuscript for important intellectual content. MR and AE participated in the interpretation of the results and in critically reviewing and revising the manuscript for important intellectual content. SS participated in the conception, design, and planning of the study, in the interpretation of the results, and in critically reviewing and revising the manuscript for important intellectual content. GB participated in the conception, design, and planning of the study, in the interpretation of the results, and in the drafting of the manuscript and critically reviewing and revising it for important intellectual content. All authors contributed to the article and approved the submitted version.

## Funding

This study and preparation of this manuscript were funded by Merck Sharp & Dohme LLC, a subsidiary of Merck & Co., Inc., Rahway, NJ, USA.

## Conflict of interest

JC, CM, and ST are employed by RTI Health Solutions, which received funding for the conduct of this study. AB-A, BM, JV, and DB are employees of MSD, Belgium. GB is an employee of MSD, Spain. AE is an employee of Merck Sharp & Dohme LLC, a subsidiary of Merck & Co., Inc., Rahway, NJ, USA. MN was an employee of Merck Sharp & Dohme LLC, a subsidiary of Merck & Co., Inc., Rahway, NJ, USA, while this study was conducted. GB, AE, and MN are shareholders in Merck & Co., Inc., Rahway, NJ, USA. SS has been involved in research and advisory boards related to the economic evaluation of vaccines funded by MSD, GSK, and Pfizer. ND reports personal fees from Roche and Boehringer-Ingelheim and nonfinancial support from Pfizer, Janssen, and MSD outside the submitted work; ND also has been involved in research related to the economic evaluation of vaccines with MSD and Pfizer as an unpaid consultant. PL has received consulting fees for advisory board participation from Sanofi. OE has received consulting fees for advisory board participation and for support of model design in the evaluation of vaccines from MSD, Pfizer, GSK, and Sanofi. JL’s employer (KU Leuven) has received an hourly wage compensation for participation in workshops related to the economic evaluation of vaccines funded by MSD and Pfizer.

## Publisher’s note

All claims expressed in this article are solely those of the authors and do not necessarily represent those of their affiliated organizations, or those of the publisher, the editors and the reviewers. Any product that may be evaluated in this article, or claim that may be made by its manufacturer, is not guaranteed or endorsed by the publisher.
